# N^6^-methyladenosine contributes to cellular phenotype in a genetically-defined model of breast cancer progression

**DOI:** 10.18632/oncotarget.25782

**Published:** 2018-07-27

**Authors:** Nate J. Fry, Brittany A. Law, Olga R. Ilkayeva, Kristen R. Carraway, Christopher L. Holley, Kyle D. Mansfield

**Affiliations:** ^1^ Biochemistry and Molecular Biology Department, Brody School of Medicine, East Carolina University, Greenville, NC 27834, USA; ^2^ Department of Medicine, Duke University Medical Center, Durham, NC 27710, USA; ^3^ Duke Molecular Physiology Institute, Duke University, Durham, NC 27701, USA

**Keywords:** N^6^-methyladenosine, hypoxia, breast cancer, transformation, RNA modification

## Abstract

The mRNA modification N^6^-methyladenosine (m6A) is involved in many post-transcriptional regulatory processes including mRNA stability and translational efficiency. However, it is also imperative to correlate these processes with phenotypic outputs during cancer progression. Here we report that m6A levels are significantly decreased in genetically-defined immortalized and oncogenically-transformed human mammary epithelial cells (HMECs), as compared with their primary cell predecessor. Furthermore, the m6A methyltransferase (METTL3) is decreased and the demethylase (ALKBH5) is increased in the immortalized and transformed cell lines, providing a possible mechanism for this basal change in m6A levels. Although the immortalized and transformed cells showed lower m6A levels than their primary parental cell line, overexpression of METTL3 and METTL14, or ALKBH5 knockdown to increase m6A levels in transformed cells increased proliferation and migration. Remarkably, these treatments had little effect on the immortalized cells. Together, these results suggest that m6A modification may be downregulated in immortalized cells as a brake against malignant progression. Finally, we found that m6A levels in the immortalized and transformed cells increased in response to hypoxia without corresponding changes in METTL3, METTL14 or ALKBH5 expression, suggesting a novel pathway for regulation of m6A levels under stress.

## INTRODUCTION

For the United States in 2018, it is estimated that there will be over 250,000 new cases of breast cancer, resulting in 40,000 deaths [1]. Although the 5-year survival rate for patients diagnosed with localized breast cancer is nearly 99%, patients who present with stage IV metastatic breast cancer have a less than 30% survival rate [2, 3]. Gene expression profiles of breast cancer have been extensively studied, but they provide an incomplete picture of the biology driving this progression from localized, low-risk disease to aggressive metastatic phenotypes. New investigations of post-transcriptional and translational pathways that regulate gene expression in breast cancer cells are therefore needed to identify novel therapeutic targets for suppressing growth and metastasis of tumor cells.

Recently, the mRNA modification N^6^-methyladenosine (m6A) has been shown to be involved in post-transcriptional gene regulation and cancer progression. Decreases in m6A levels have been directly correlated to metastasis and poor patient prognosis in hepatocellular carcinoma [4] and disease progression in cervical cancer [5]. However, experimental models focused on manipulating m6A levels have given conflicting results regarding the role of m6A in malignant progression. On one hand, loss of m6A through an increase in ALKBH5, an m6A demethylase, led to enhanced breast and glioblastoma cancer stem cell self-renewal and growth [6–8]. On the other hand, an increase in m6A driven by overexpression of the methyltransferase, METTL3, led to increased invasion of lung adenocarcinoma cells [9], but inhibited growth of renal cell carcinoma cells [10]. Additionally, METTL3 has also been shown to promote translation in human lung cancer cells independent of its m6A methylation activity [9]. As the roles for m6A modifications in cancer begin to emerge, it will be important to understand the specific role that the m6A modification plays in different cancer types and at different stages in the development of tumors.

The m6A modification is the most abundant modification in mRNA [11]. This modification has been shown to be important for the stability and translational efficiency of mRNA [12–17], and is involved in the pluripotency of stem cells in embryonic development [18–20]. Methyltransferase-like (METTL)-3 and -14, as well as Wilms’ tumor associating protein (WTAP) form the m6A methyltransferase complex which modifies nascent pre-mRNA within the nucleus [21–25]. The enzymatically active component of the methyltransferase, METTL3, contains an S-adenosyl methionine (SAM) binding domain, and utilizes SAM as a substrate to methylate target mRNAs that contain a DRACH m6A consensus sequence, often found in 3′ UTR's and around both start and stop codons [21, 26–29]. METTL14 lacks catalytic activity but participates in mRNA binding/targeting [30–32]. WTAP is responsible for the localization of the METTL3/14 complex to the nuclear speckle, and greatly enhances methyltransferase activity by bringing the methyltransferase to the pre-mRNA [21, 22]. m6A methylation of mRNA can be reportedly removed by alkylation repair homolog 5 (ALKBH5) and fat mass and obesity related protein (FTO). Interestingly, a recent study suggests that FTO can also demethylate *N*^6^,2′-*O*-dimethyladenosine (m6Am) raising questions about its preferred *in vivo* substrate [33].

Once an mRNA is methylated, it can be bound by the YTH family of RNA binding proteins, including YTHDF1, YTHDF2, and YTHDC1 [15, 34, 35]. The broader consequences of RNA methylation through the actions of these and other m6A RNA binding proteins are still being investigated. However, YTHDF2 has been shown to facilitate degradation of methylated mRNAs by transporting them to P bodies [15, 36–38]. Alternatively, binding of YTHDF1 increases translational efficiency of m6A methylated mRNA [16]. Lastly, YTHDC1 recruits splicing factors to regulate splicing of m6A methylated mRNA [39]. The interactions between these RNA binding proteins is not fully understood, and competition between them may yield different fates for the methylated mRNA and ultimately for the protein output.

As mentioned previously, m6A methylation of RNA has recently been correlated with a number of phenotypic changes in a variety of cancers including breast cancer [4–10]. Many of these phenotypic changes are the result of changing protein expression of either the m6A methyltransferases, demethylases or RNA binding proteins. These studies have shown that m6A has a functional significance in cancer, but there remain incomplete connections between m6A modifications and cancer cell phenotypes. For example, tumors can quickly outgrow their blood supply during cancer progression and they therefore must adapt to hypoxic conditions. Hypoxic breast cancer cells adapt to these conditions through Hypoxia Inducible Factor (HIF)-mediated angiogenesis [40]. Not only does HIF increase vascularization of the tumor to increase blood and oxygen supply, but it is also known to promote metastasis of the cells [41–43]. Interestingly, ALKBH5, an m6A demethylase, is also regulated by HIF [44]. Recently, it was reported that a HIF-regulated decrease in m6A through an increase in ALKBH5 and/or ZNF217 expression maintains pluripotency of breast cancer stem cells in several established breast cancer cell lines [7, 45]. Furthermore, we recently reported that hypoxia led to an increase in m6A mRNA levels in HEK-293T cells, leading to increased stability and recovery of translational efficiency after re-oxygenation [17]. Because hypoxia both regulates m6A levels and promotes metastasis in breast cancer cells, it is important to understand if m6A might have a role in hypoxia- induced breast cancer metastasis.

Our current study aimed to define the landscape of m6A modification during breast cancer development and progression. Because cancers have many diverse mutations and alterations to gene regulation, it has been difficult to pinpoint exactly which changes introduce aggressive phenotypic behavior. For this reason, we chose to use a genetically-defined breast cancer progression model for these studies. In this model, three cell types are utilized: primary Human Mammary Epithelial cells (HMECs), HMECs immortalized through the stable expression of hTERT, p53^DD^, cyclin D1, CDK4^R24C^, and C-MYC^T58A^, and a further transformed line expressing with H-RAS^G12V^ in addition to the above alterations (Supplementary Figure 1) [46, 47].

Using this model of breast cancer development, we found that immortalization resulted in reduced m6A levels as well as significant down-regulation of the m6A methylation complex (METTL3/14) and up-regulation of the primary demethylase (ALKBH5). These modifications were maintained, but not enhanced, during malignant transformation. Experimentally increasing the level of m6A modification led to a more malignant phenotype in transformed cells, but not their immortalized precursors. Finally, we found that stress from hypoxia stimulated an increase in m6A levels in both immortalized and transformed cells through a pathway that is independent of METTL3/14 and ALKBH5 expression levels, but reliant on HIF. Surprisingly, increasing m6A levels led to a more malignant phenotype in transformed cells, but not their immortalized precursors.

## RESULTS

**Figure 1:**
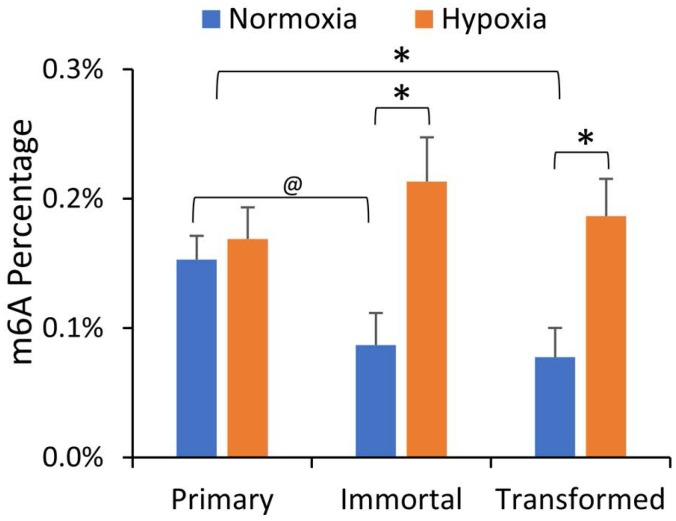
mRNA m6A levels are decreased in immortalized and transformed HMECs but increased by hypoxia LC-MS/MS of mRNA nucleosides isolated from primary, immortalized, and transformed HMECs grown in normoxia or hypoxia (1% O_2_) for 24 hours. Values represent the amount of m6A relative to total Adenosine content. ^*^P ≤ 0.05, ^@^*P* ≤ 0.1 by paired Student's *t*-test. Error bars represent standard error of the mean (SEM) of 3 experiments.

We first investigated whether m6A levels were altered in our breast cancer progression model, and what effect hypoxia had on mRNA m6A content in the HMEC cell lines. HMEC cells (primary, immortalized, and transformed) were incubated for 24 hours under normoxic or hypoxic (1% O_2_) conditions. PolyA^+^ mRNA was isolated by oligo-dT selection followed by ribosomal RNA (rRNA) depletion, and after digestion to individual nucleotides, ultra-high-performance liquid chromatography and tandem mass spectrometry (UPLC-MS/MS) were used to quantify various RNA modifications in the mRNA enriched samples. As shown in Figure 1, normoxic m6A levels were decreased in both the immortalized and transformed cell lines in comparison to the primary HMECs. While hypoxia had no effect on the m6A level in the primary cells, it significantly increased m6A levels in immortalized and transformed cells (Figure 1). This is consistent with our prior report that hypoxia raises m6A levels in the transformed HEK293T cell line [17]. Other mRNA modifications, including 5-methylcytidine, remained unchanged in this model of breast cancer progression or were not influenced by hypoxia, (Supplementary Figure 2), suggesting that m6A levels are particularly important in the progression to oncogenic transformation and the hypoxia response.

### Protein levels of m6A methyltransferases and demethylases

**Figure 2:**
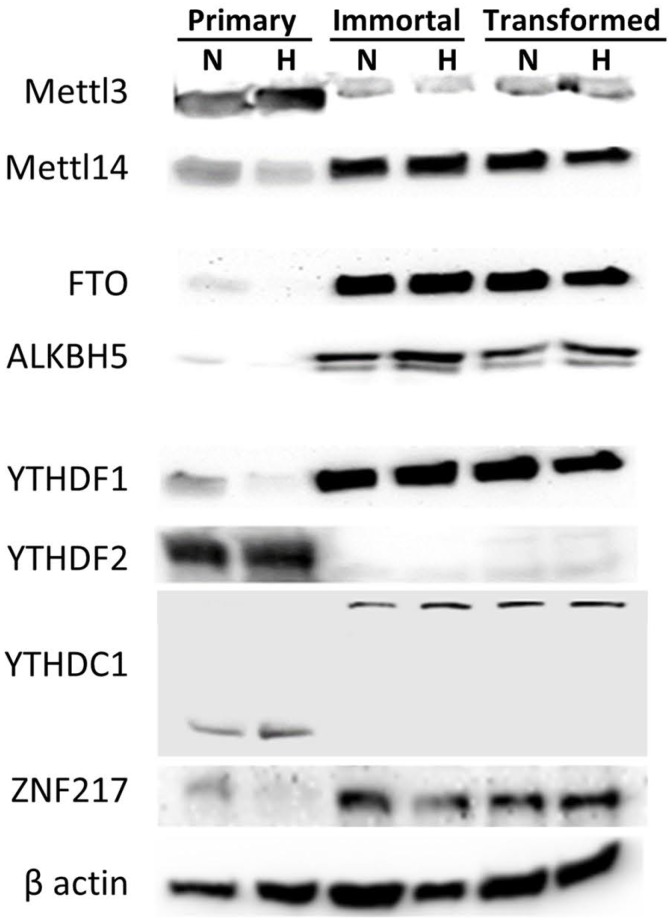
Protein expression of m6A methyltransferases, demethylases and RNA binding proteins changes during HMEC immortalization/transformation Western blots of 50 μg of protein lysates from normoxic (N) or hypoxic (H) primary, immortalized, and transformed HMECs. (Representative of 3 experiments).

To investigate the dynamic response of m6A to cellular transformation as well as hypoxia, we measured RNA and protein levels of the m6A-associated enzymes and effector proteins involved with m6A, including methyltransferases, demethylases, and RNA binding proteins, many of which exhibited notable changes upon immortalization and oncogenic transformation of the HMEC cells (Figure 2). Protein and RNA expression for the enzymatically active subunit of the methyltransferase, METTL3, was decreased in immortalized and transformed cells, but METTL14 was increased (Figure 2, Supplementary Figure 3). In addition, ZNF217 protein, which is known to sequester METTL3 and prevent its methyltransferase activity [48], is also increased in the immortalized and transformed cells. RNA and protein expression for demethylases ALKBH5 and FTO were also increased in the immortalized and transformed cells, which is concordant with the decreased m6A levels in these cells. This data suggests that the loss of m6A observed in the transformed cells may be due to an increase in ZNF217, a loss of the methyltransferase, METTL3, and an increase of the demethylases, ALKBH5 and FTO. Hypoxia, however, had very little effect on the protein levels. Therefore, the increase in m6A levels in response to hypoxia is not explained by the observed protein levels of the methyltransferases or demethylases, suggesting that it must be due to some other process.

The m6A binding proteins YTHDF1, YTHDF2 and YTHDC1 were also altered in the immortalized and transformed cells lines. YTHDF1 levels were increased, but YTHDF2 levels were decreased in the immortalized and transformed cells. The blots for YTHDC1 interestingly contained a shift from a lower molecular weight (the correct expected molecular weight) to a much higher molecular weight (the observed molecular weight given in the details of the antibody). Given the known roles of YTHDF1 and 2 [12, 16], the switch from high YTHDF2 and low YTHDF1 expression to high YTHDF1 and low YTHDF2 suggests that m6A modifications in the immortalized and transformed cells are no longer primarily directing degradation of mRNA, but rather increasing translational efficiency. Notably, RNA expression of YTHDF2 was strongly increased in immortal and transformed cells, despite the decreased protein levels (Supplementary Figure 3).

### Differential methylation of specific mRNAs in response to hypoxia

**Figure 3:**
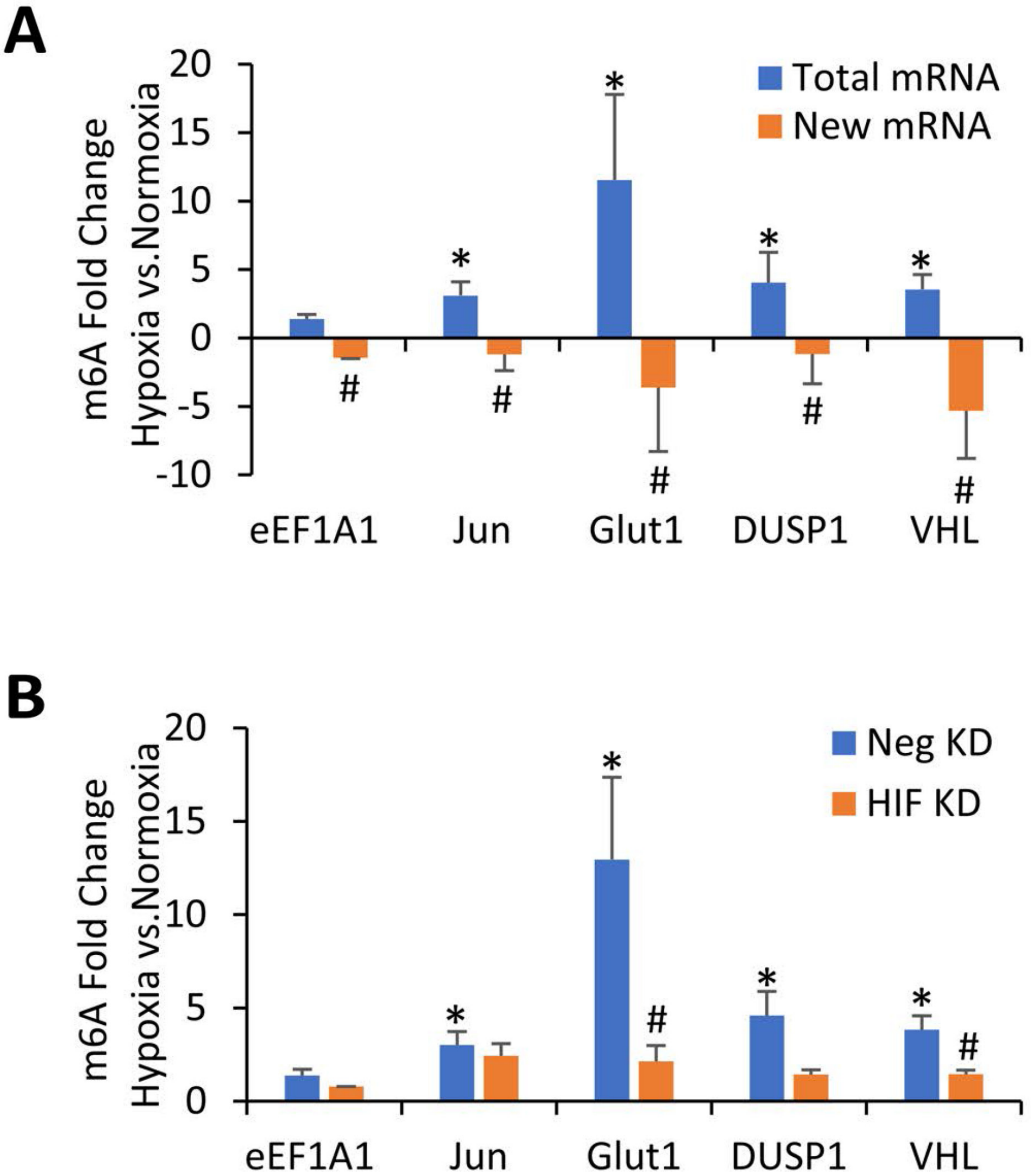
m6A methylation of specific mRNAs increases in transformed HMECs exposed to hypoxia in a HIF dependent manner (**A**) MeRIP of 100 ng of total mRNA (Blue Bars) or newly transcribed mRNA (Orange Bars) from transformed HMECs. Cells were grown in normoxic or hypoxic conditions for 24 hours and mRNA was quantified by qRT-PCR. Fold enrichments calculated from immunoprecipitated RNA levels normalized to input RNA and expressed as a ratio of hypoxia/normoxia. ^*^P ≤ 0.05 by unpaired Student's *t*-test represents increased m6A levels in hypoxia compared to normoxia. ^#^*P* ≤ 0.05 represents a decrease in m6A in newly transcribed RNAs in hypoxia as compared to total RNAs in hypoxia. Error bars represent SEM of 3 experiments. (**B**) MeRIP of 100 ng of mRNA from transformed HMECs. Cells were transfected with control (Neg KD) or HIF-1α and HIF-2α siRNA (HIF KD) and grown for 48 hours before being exposed to either normoxic or hypoxic conditions for 24 hours. RNA was quantified by qRT-PCR. Fold enrichments calculated from immunoprecipitated mRNA levels normalized to input RNA and expressed as a ratio of hypoxia/normoxia. ^*^P ≤ 0.05 by unpaired Student's *t*-test represents increased m6A levels in hypoxia compared to normoxia. ^#^*P* ≤ 0.05 between control and HIF KD by unpaired Student's *t*-test. Error bars represent SEM of 4 experiments.

Although protein expression levels of the m6A methyltransferases and demethylases could not explain why m6A is increased in hypoxia for the immortalized and transformed HMECs, we have previously shown that m6A itself can stabilize certain mRNAs under hypoxic conditions, including Glut1, Jun, Myc, and DUSP1 [17]. Therefore, it is possible that the hypoxic increase in m6A levels is simply due to a relative accumulation of mRNAs that have been stabilized by m6A modifications. In order to test this possibility, we used m6A RNA immunoprecipitation (MeRIP) to assay previously-identified mRNA targets for increased m6A levels after 24 hours of hypoxic conditions [17]. This approach allows for quantification of changes in m6A content by normalizing the amount of MeRIP-captured RNA to the amount of input RNA for each sample, determining a percentage of m6A methylated mRNA for each species. These percentages can then be compared between different conditions (normoxia and hypoxia) to determine the effect of those conditions on m6A levels in specific mRNAs. In the transformed HMEC line, upon exposure to 24 hours of hypoxia, m6A content was significantly increased in transcripts for Glut1, Jun, VHL, and Dusp1, but not eEF1A1 (Figure 3A, blue bars). These results extend our previous findings in 293T cells, which also showed that these transcripts have increased m6A content in response to hypoxia [17]. MeRIP from immortalized cells showed a similar trend, but primary HMEC cells showed minimal differences in m6A levels for these transcripts (Supplementary Figure 4). These data demonstrate a specific increase in the m6A of these mRNAs from transformed HMECs in response to 24 hours hypoxic exposure.

We next tested whether 24 hours of hypoxic exposure resulted in increased methylation of newly transcribed RNA in the transformed HMECs. A uridine analog, 4-thiouridine (4sU), was added to the culture media for the final hour of exposure, to be incorporated into all newly transcribed RNA. After isolation, this newly transcribed RNA was biotinylated and separated from old (unlabeled) mRNA using streptavidin beads. After elution of the newly transcribed RNA from the streptavidin beads, MeRIP was then used to pull down m6A methylated RNA from this population of new RNA. In contrast to the total mRNA, newly transcribed mRNA had less m6A methylation in hypoxia than normoxia (Figure 3A, orange bars), suggesting that methylation of these newly transcribed RNAs was not increased at 24 hours of hypoxic exposure. This data then also suggests that the increase in m6A seen after 24 hours of hypoxic exposure was not due to a sustained increase in the methylation of newly transcribed mRNA, but more likely an accumulation of pre-existing, methylated mRNAs.

### HIF controls hypoxic m6A levels in specific targets

To further investigate how m6A was increased under hypoxia, we wanted to determine if the HIF transcriptional response was involved. HIF-1α and HIF-2α were simultaneously knocked down via siRNA in transformed cells which were then exposed to 24 hours of hypoxia. Knockdown of HIF-1α and HIF-2α was confirmed via western blot analysis (Supplementary Figure 5). m6A content in individual mRNAs were measured once again by MeRIP followed by RT-qPCR. Knockdown of HIF-1α and HIF-2α prevented the hypoxic increase in m6A levels in many of our targets including Glut1, VHL, and Dusp1 (Figure 3B). This suggests that the increase in m6A in hypoxia is at least partially due to the hypoxic induction of HIF.

### Phenotypic effect of m6A modulation

**Figure 4:**
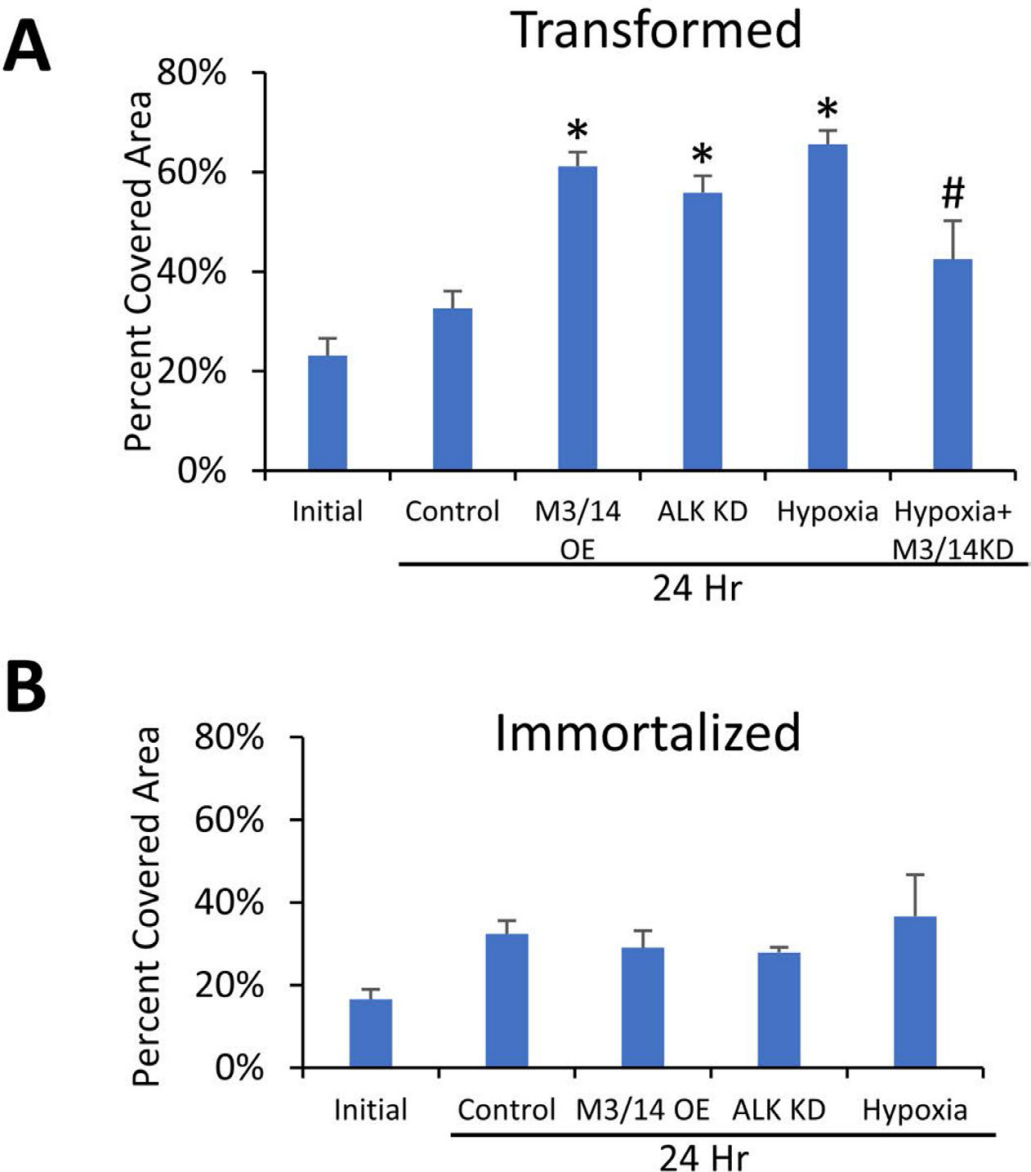
m6A levels impact cellular migration in transformed, but not immortalized HMECs Wound healing scratch assay of transformed (**A**) and immortalized (**B**) HMECs. HMECs were transfected with siRNA targeting METTL3 and METTL14 (M3/14 KD), plasmids overexpressing METTL3 and METTL14 (METTL3/14 OE), plasmid expressing ALKBH5 shRNA (ALK KD), or control siRNAs/plasmids and allowed to grow to confluency. After 8 hours of serum starvation, a scratch was made with a p200 tip, cells were washed and initial pictures (0 Hr) taken. Cells were then incubated in serum free media in either normoxic or hypoxic conditions for 24 hours before a second set of pictures was taken (24 Hr). Migration was quantified by measuring the area of cell coverage of three representative fields at 0 and 24 hours. ^*^P ≤ 0.05 by Unpaired Student's *t*-test. Error bars represent SEM of 3–4 experiments.

We then sought to determine the differences in m6A levels between progressive stages of a genetically-defined breast cancer model, but also to understand if changes in m6A levels during pre-malignant immortalization of primary cells has a functional role in regulating the response to hypoxia. Because hypoxia has been reported to induce cell migration, we measured changes in migratory potential in immortalized and transformed cells in response to hypoxia. Exposure of the transformed HMECs to 24 hours of hypoxia led to increased cell migration when compared to normoxia (Figure 4A), but surprisingly had little effect on migration of the immortalized cells (Figure 4B). Simultaneous knockdown of METTL3 and METTL14 via siRNA in the transformed cells significantly reduced the hypoxic increase (Figure 4A), suggesting that m6A is directly involved in this phenotypic change. Furthermore, increasing m6A levels by simultaneous overexpression of the m6A methyltransferases METTL3 and 14 or by knockdown of the demethylase ALKBH5 (Figure 4A) in normoxic conditions also led to an increase in migration of the transformed cells, further supporting a role for increased m6A in this phenotypic change. In contrast to the transformed cells, no significant differences were seen in the migration of the immortalized cells in any of these conditions when compared to controls (Figure 4B). Confirmation of METTL3/14 overexpression and ALKBH5 knockdown is shown in Supplementary Figure 5 and representative scratch assay images can be found in Supplementary Figure 6.

**Figure 5:**
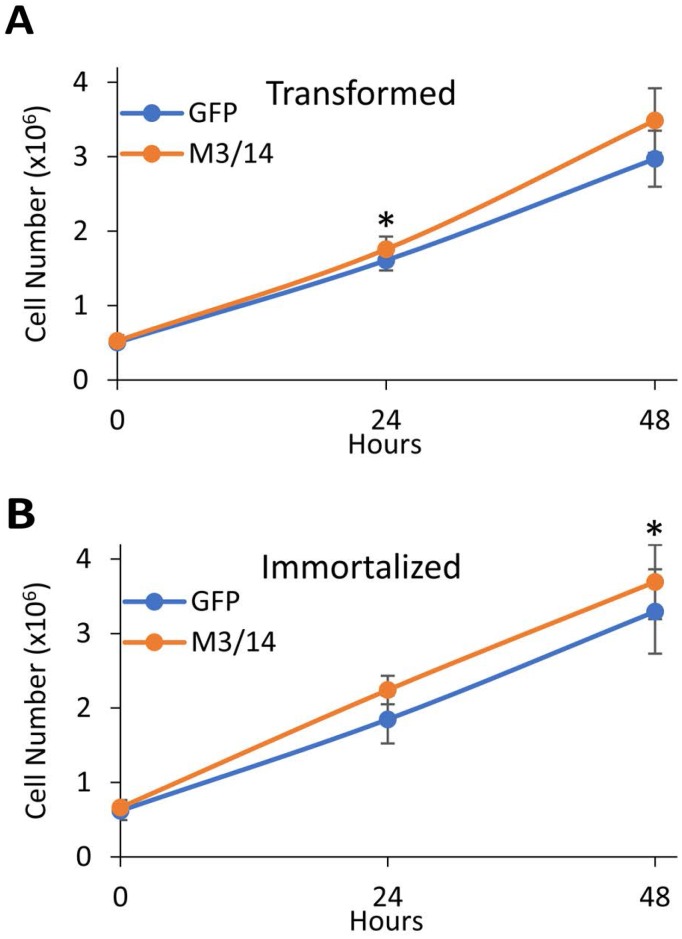
METTL3 and 14 overexpression leads to increased proliferation of transformed and immortal HMECs Transformed (**A**) and immortalized (**B**) HMECs were transfected with either a control GFP expressing plasmid or plasmids overexpressing METTL3 and METTL14 (M3/14). 48 hours after transfection, cells were re-plated at 0.5 × 10^6^ cells/well of a 6 well plate and allowed to recover for 16 hours. Cells were then detached by trypsin at each time point (0, 24, or 48 hours), and counted in a hemocytometer to determine cell number. ^*^P ≤ 0.05 by paired Student's *t*-test. Error bars represent SEM of 3 experiments.

Interestingly, overexpression of METTL3 and 14 increased proliferation in both the transformed (Figure 5A) and immortalized (Figure 5B) cells. Although the effect was not robust and only significant at specific time points for each cell line, it again suggests that increases in cellular m6A levels can alter the phenotypes of the breast cancer cells.

### Summary

Our findings indicate that mRNA m6A methylation levels decrease during immortalization of primary HMECs. Consistent with our previous findings, hypoxia increases mRNA m6A levels in both immortalized transformed cells [17]. These increases in m6A in hypoxia are regulated by HIF, and are not due to increased protein levels of the methyltransferase or methylation rates. Increasing m6A methylation by overexpressing methyltransferases and/or hypoxic exposure in the transformed cells, increased proliferation and migration, while only affecting proliferation in the immortalized cells, indicating that pre-malignant and malignant cells may utilize m6A to in different ways.

## DISCUSSION

Although gene expression in breast cancer has been extensively studied, the contribution of mRNA modifications to breast cancer progression is not known. An advantage to using a genetically-defined breast cancer model is that it limits the heterogeneity that is inherent to patient-derived samples which may aid in uncovering the mechanisms underlying the phenotypic changes driven by increased m6A. Studies involving RNA modifications, including the m6A modification, may lead to a better understanding of gene regulation in cancers.

In this study, we show that the m6A modification of mRNA decreases after immortalization and subsequent oncogenic transformation of primary HMECs maintained under normoxia. These results provide a more detailed view of cancer progression, and support prior findings that low levels of m6A modification correlate with a more oncogenic or aggressive cellular phenotype. However, our results also show that hypoxia drives m6A modification of mRNA back to primary cell levels. Our findings differ from previous reports that have shown total RNA m6A levels in breast cancer cell lines decrease in hypoxia conditions through HIF mediated induction of the demethylase ALKBH5 and/or METTL3 sequestration by ZNF217 [7, 45]. These reports indicated that hypoxia induces a breast cancer stem cell phenotype through the decrease in m6A. The results seen here differ from these previous reports possibly due to differences in model systems, and hence expressed genes. For example, the previous reports show that m6A levels in specific mRNAs, including Nanog, is decreased in hypoxia. However, Nanog mRNA is not highly expressed in our cell lines and no differences in its m6A levels were observed (data not shown). In addition, the previous studies also used total RNA rather than mRNA to measure differences in global m6A levels, so changes in noncoding RNA species including ribosomal RNA may explain the differences in those studies.

To understand the mechanism by which m6A levels are regulated in our model system, protein expression of the methyltransferase, demethylases, and m6A RNA binding proteins were measured. Interestingly, the enzymatically active subunit of the methyltransferase complex, METTL3, is decreased in the immortalized and transformed cells, and ZNF217 which sequesters METTL3 is increased. Additionally, the two known m6A demethylases, ALKBH5 and FTO, increase in the immortalized and transformed cells. Therefore, it is possible that the decrease in m6A methylation in the immortalized and transformed cells may be due to a loss in methylation potential and an increase in demethylation potential. In future experiments, it would be interesting to investigate which of these expression changes are necessary and/or sufficient for the m6A changes observed.

In addition to effects on the m6A enzymes, protein expression of m6A specific RNA binding proteins also change in the immortalized and transformed cells. YTHDF2, an m6A RNA binding protein which leads to degradation of the methylated RNA [15, 36], decreased in the immortalized and transformed cells. In contrast, YTHDF1, another m6A specific RNA binding protein involved in methylated mRNA translational efficiency increases in the immortalized and transformed cells [16]. These contrasting changes in RNA binding proteins suggest that the remaining methylated RNA have increased translational efficiency through increased YTHDF1 levels and an increase in stability due to a decrease in YTHDF2 levels. The RNA binding protein YTHDC1 interestingly appears expressed at a higher molecular weight in the immortalized and transformed cells. This may possibly be due to dimerization of YTHDC1, however this has not been confirmed.

We also observed that hypoxia did not lead to changes in any of the aforementioned proteins. Because the increase of m6A in hypoxia cannot be explained by protein levels of methyltransferases, demethylases, or ZNF217, we sought an alternative mechanism for this change. Our group has previously reported that increased m6A methylation in hypoxia leads to stabilization of mRNAs [17]. Therefore, it is possible that the increase in m6A methylation is due to stabilization and accumulation of methylated mRNAs. It is also possible that upon hypoxic exposure methylation rates of newly transcribed RNA are increased or that increased methylation (or decreased demethylation) of mature mRNA is occurring within the cytoplasm, as has recently been shown in neuronal cells [49]. However, a recent study has shown that m6A methylation is not dynamic within the cytoplasm, which would suggest that neither methylation nor demethylation levels are altered on mature mRNA [25].

We investigated whether prolonged hypoxic exposure increased the methylation of newly transcribed RNA. These experiments showed that after 24 hours of hypoxia, methylation of newly transcribed RNA was not increased, and in fact trended towards a decrease of m6A in newly transcribed RNA. Given this observation and our previous findings that m6A containing mRNA are stabilized under hypoxia, it seems more likely that the increase in m6A levels is due to the accumulation of the stabilized mRNA. This scenario still leaves open the possibility of an increase in newly transcribed RNA methylation earlier in the hypoxic exposure that then returns to baseline or below by 24 hours. However, even in this scenario, the increased m6A levels in those RNAs are likely maintained through the stabilization of those mRNAs, a possibility that we are currently exploring.

Because the hypoxic response through HIF is crucial for survival and tumorigenesis of cancer cells under hypoxic conditions, the effect of HIF on m6A levels in hypoxia was measured. Previous reports using breast cancer stem cells have shown that hypoxic activation of HIF led to decreased RNA methylation through HIF-mediated induction of the demethylase ALKBH5 and METTL3 suppressor ZNF217 [7, 45]. However, in our model system of differentiated human mammary epithelial cells there is an increase in m6A in hypoxia, rather than a decrease, and no change in either ALKBH5 or ZNF217 expression levels. Furthermore, knockdown of HIF decreased hypoxic m6A levels in many of our specific targets including Glut1, Dusp1, and VHL, suggesting that HIF transcriptional activity is involved in the increase of m6A in these targets. The conflicting data with the previous report may be explained through a difference in model system. However, it would also be informative to investigate the role of ZNF217 in our HMEC-based system to see if it interacts with METTL3 and the m6A system and whether that interaction is regulated during breast cancer progression. Indeed, these results, along with contrasting effects of m6A in other cancer types highlights the importance of understanding the m6A modification in all cancer systems at various stages of disease.

Understanding mechanisms behind m6A methylation is important in breast cancer, as it may lead to a better understanding of the cancer itself. However, it is also important to observe the phenotypic effects that m6A modulation has on breast cancer. As stated previously, immortalization and oncogenic transformation of HMEC cells led to a decrease in m6A methylation. It therefore seemed likely that an increase of m6A in these transformed cells would drive cells back toward a more primary-cell phenotype. However, increasing m6A levels either through hypoxia or METTL3/14 overexpression in the transformed cells increased proliferation and migration of those cells. Thus, it appears likely that an increase in m6A either through methyltransferase overexpression or an as of yet unidentified HIF-mediated mechanism promotes a more malignant phenotype. It is also possible that METTL3 could be acting in an m6A-independent manner as has been shown in lung cancer cells [9]. However, because ALKBH5 knockdown and hypoxia both recapitulated the METLL3/14 overexpression results, it would suggest that it is the increase in m6A itself that was underlying the phenotypic changes. Interestingly, the immortalized cells did not show the same response as transformed cells, suggesting that the increased m6A was working in tandem with expression of oncogenic HRasG12V (the only transgene to differ between the two cell lines) in order to alter these phenotypes. Future studies will investigate this relationship between Ras transformation and the role of m6A in promoting tumor phenotypes.

Overall, this study demonstrates that m6A methylation is important for the phenotypic progression of breast cancer in a genetically-defined model. Hypoxia appears to play a role in this progression through increases in mRNA m6A levels in breast cancer through HIF activation. This hypoxic response can be mimicked in normal cells by experimentally raising m6A levels. While it is clear m6A plays a role in these processes, it is imperative that we now identify the mRNA that are being affected and determine the impact on gene expression in order to understand the underlying biological causes for these phenotypic changes.

## MATERIALS AND METHODS

### Cell lines

HMEC Primary cell lines cells were obtained directly from Lonza (Walkersville, MD) and maintained in Mammary Epithelial Basal Medium (MEBM) (Lonza) and supplemented with Mammary Epithelial Cell Growth Medium (MEGM) BulletKit (Lonza) along with 2 mM Glutamine (Corning/Mediatech), and 1× Pen/Strep (Corning/Mediatech) and passaged when approximately 85–90% confluent. Immortalized and transformed cells were gifts from Jack Keene's lab [46]. Immortalized and transformed cells were maintained in MEGM+10% FBS. Cells were tested for mycoplasma upon receipt. For experiments, cells were plated on 10 cm dishes (CytoOne, USA Scientific, Orlando, FL) allowed to attach/recover for 18–24 hours. The next day, the media was removed and replaced with fresh media. Hypoxic treatments were carried out in a Ruskin *In Vivo* 400 Hypoxia Hood (The Baker Company, Sanford, ME) maintained at 37° C, 5% CO_2_, 70% humidity and 1% oxygen. All other chemical reagents were obtained from Sigma-Aldrich (St. Louis, MO) unless otherwise specified.

### RNA extraction

Trizol (Life Technologies, Carlsbad, CA) was used for all RNA extractions according to the manufacturer's protocol. RNA was further purified and treated with RNase-Free DNase I (Life Technologies) using PureLink RNA Mini Kit (Life Technologies). For RNA extraction from ribonucleoprotein immunoprecipitations (RNP-IP) and sucrose gradients, GlycoBlue (Life Technologies) was added as a carrier during the precipitation step. RNA purity and quantity was determined via NanoDrop 1000 (ThermoFisher Scientific, Waltham, MA).

### LC-MS/MS of PolyA^+^ RNA

PolyA^+^ RNA was first purified from total RNA through oligo-dT selection using a Poly(A)Purist-MAG magnetic mRNA Purification Kit (Life Technologies) followed by ribosomal RNA depletion using RiboMinus Eukaryote Kit (Life Technologies) according to the manufacturer's protocols. Purified PolyA^+^ RNA was digested to individual nucleosides and modified nucleosides were quantified as previously described [50]. Briefly, digestion was performed with nuclease P1 (Sigma, 2 U) in buffer containing 25 mM NaCl and 2.5 mM ZnCl2 for 2 h at 37° C, followed by incubation with Antarctic Phosphatase (NEB, 5 U) for an additional 2 h at 37° C. Nucleosides were then separated and quantified using UPLC-MS/MS as previously described [51], except acetic acid replaced formic acid in the mobile phase.

### Western blots

Whole cell lysates were prepared in whole cell extract buffer (WCEB: 50 mM Tris pH 7.4, 150 mM NaCl, 5 mM EDTA, 0.1% SDS, and complete protease inhibitor (Promega, Madison, WI)). Equal amounts of protein (30–50 μg) were electrophoresed on a mini-PROTEAN any KD acrylamide gel (Bio-Rad Laboratories, Hercules, CA) and transferred to Hybond ECL nitrocellulose (GE Healthcare, Chicago, IL). Transfer was verified via Ponceau S staining then blot was blocked with 5% nonfat dry milk (LabScientific, Highlands, NJ) in Tris buffered saline with 0.1% Tween 20 (TBST) for one hour at room temperature, followed by primary antibody in blocking buffer overnight at 4° C. After washing extensively with TBST, blots were incubated for 1–2 hours at room temperature with appropriate anti-mouse (GE Healthcare), anti-rabbit (GE Healthcare), or Rabbit anti-goat (Novus Biologicals, Littleton, CO), washed again with TBST, detected using Bio-Rad Clarity Western ECL Substrate (Bio-Rad Laboratories), and imaged via _MY_ECL Imager (Thermo Scientific). Primary Antibodies used and their concentrations can be found in Supplementary Table 3.

### m6A mRNA immunoprecipitation (MeRIP)

m6A Ribonucleoprotein Immunoprecipitation reactions were performed by first isolating PolyA^+^ RNA from normoxic and hypoxic cells. Protein G Dynabeads (Thermo Fisher Scientific, Baltics UAB) were washed 3× in 1 mL of IPP buffer (10 mM Tris-HCL pH7.4, 150 mM NaCl, 0.1% NP-40). 25 μl of beads required per IP. Anti-N^6^-methyladenosine mouse monoclonal antibody (EMD Millipore, Temecula, CA, MABE1006) was added to the beads (5 μg/IP) and brought up to 1mL with IPP buffer. Bead mixture was tumbled for 16 hours at 4° C. Beads were washed 5× with IPP buffer and 100 ng of PolyA^+^ RNA was added to the beads along with 1 mM DTT and RNase out. The mixture was brought up to 500 μl with IPP buffer. Bead mixture was tumbled at 4° C for 4 hours. Beads were washed 2× in IPP buffer, placed in to a fresh tube, and washed 3× more in IPP buffer. m6A RNA was eluted off the beads by tumbling 2× with 125 μl of 2.5 mg/mL N^6^-Methyladenosine-5′-monophosphate sodium salt (CHEM-IMPEX INT'L INC., Wood Dale, IL). Supernatant was added to Trizol-LS followed by RNA isolation as per manufacture's protocol. Final RNA sample was brought up in 10 μl of water.

### PCR for MeRIP

Reverse transcription was performed on 10 μl m6A PolyA^+^ RNA from the MeRIP with the iScript cDNA synthesis kit (Bio-Rad Laboratories, Hercules, CA). After diluting cDNA two-fold, quantitative real-time PCR was performed using a Roche Lightcycler 96 with Fast Start Essential DNA Green (Roche Diagnostics Corporation, Indianapolis, IN) and primers from Integrated DNA Technologies, Inc. (Coralville, Iowa). Primers used are listed in Supplementary Table 1. Primer efficiency was verified to be over 95% for all primer sets used. Quantification of mRNA from the MeRIP was carried out via ΔCT analysis against non-immunoprecipitated input RNA. All real-time PCR primer sets were designed so the products would span at least one intron (>1 kb when possible), and amplification of a single product was confirmed by agarose gel visualization and/or melting curve analysis.

### siRNA transfections

Either a negative siRNA (Silencer; Life Technologies, Carlsbad, CA) or HIF-1α and HIF-2α siRNAs or METTL3 and METTL14 siRNAs (Qiagen, Germantown, MD) were transfected using Lipofectamine RNAi Max per manufacturer's protocol (Life Technologies). siRNAs used can be found in Supplementary Table 2. Cells were incubated for 72 hours post-transfection with the last 24 hours in either normoxic or hypoxic conditions as indicated.

### Plasmid transfections

Either a negative control plasmid, shRNA scramble control, ALKBH5 psi-U6 shRNA construct (GeneCopoeia, Rockville, MD) or a METTL3 and METTL14 flag tagged construct given by Dr. Jing Crystal Zhao [19] was transiently transfected in immortalized and oncogenically transformed HMEC cells using Lipofectamine 2000 (Life Technologies) in 6 well plates (USA Scientific). Cells were incubated for 48 hours post-transfection before proliferation or scratch assays.

### Scratch assays

48 hours after transfection, cells were serum starved 8 hours prior to the scratch. Afterwards, a scratch was made with a p200 pipette tip (USA Scientific), and cells were washed 2× with Dulbecco's Phosphate Buffered Saline (DPBS) (Corning) and fresh serum free media added. Pictures were taken at 0 and 24 hours and wound healing determined by measuring the percentage of the visible area that was covered by cells.

## SUPPLEMENTARY MATERIALS FIGURES AND TABLES



## Abbreviations

m6A: N^6^-methyladenosine
HMECs: human mammary epithelial cells
rRNA: ribosomal RNA
LC-MS/MS: liquid chromatography and tandem mass spectrometry
MeRIP: m6A RNA immunoprecipitation
4sU: 4–thiouridine
