# Supplementary material for: N6-methyladenosine contributes to cellular phenotype in a genetically-defined model of breast cancer progression
Source: Oncotarget. 2018 Jul 27;9(58):31231–43. doi: 10.18632/oncotarget.25782 (PMC6101291; doi:10.18632/oncotarget.25782)
Supplement: Supplementary file 1 [file oncotarget-09-31231-s001.pdf]

N^6-methyladenosine contributes to cellular phenotype in a genetically-defined model of breast cancer progression

SUPPLEMENTARY MATERIALS

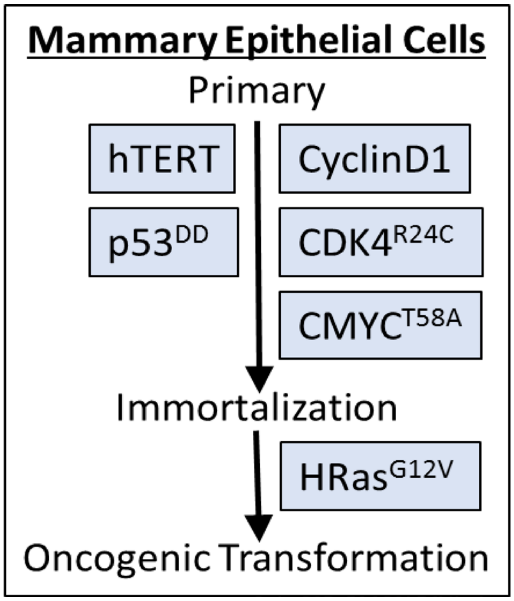

Supplementary Figure 1: Outline of the cellular transformation in the genetically defined human mammary epithelial cancer cells.

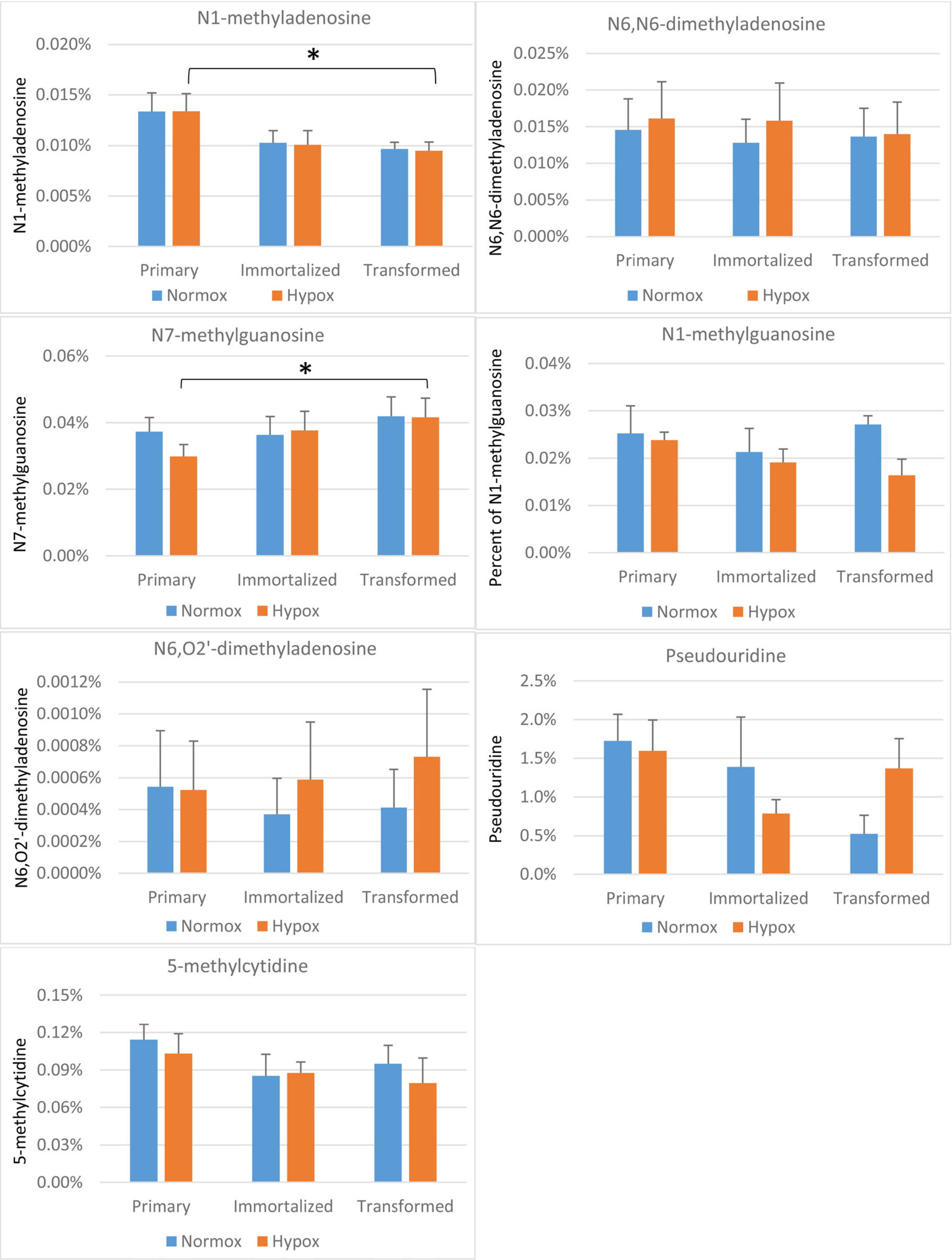

Supplementary Figure 2: UPLC-MS/MS of Poly A^+ RNA. HMEC cells cultured in Normoxic or Hypoxic conditions for 24 hours. Values represent the amount of the modification divided by unmodified levels ($n = 3$). $^*P \le 0.05$ by paired Student's t-test. Error bars represent standard error of the mean (SEM).

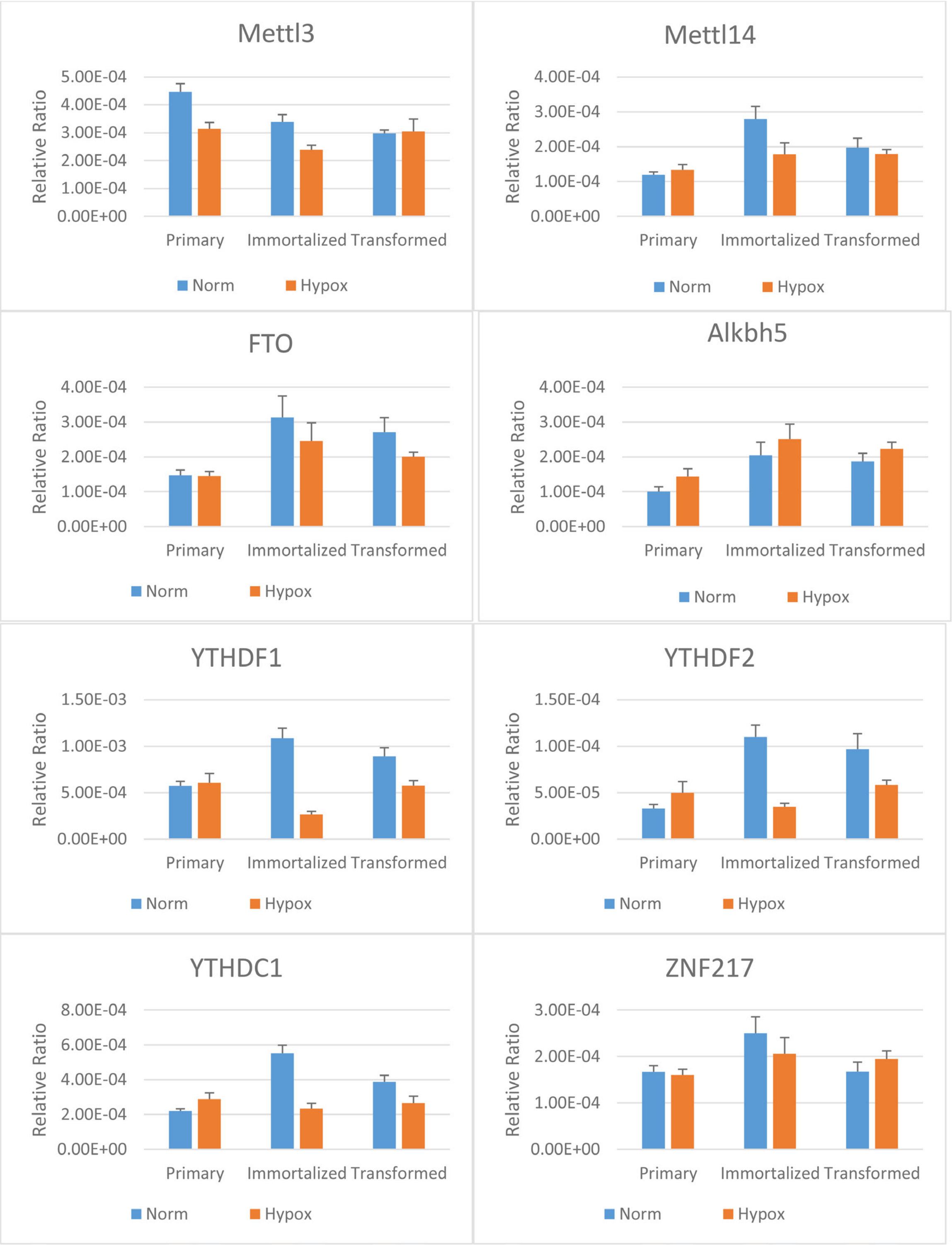

Supplementary Figure 3: Relative RNA expression levels. qRT-PCR of m6A methyltransferases, demethylases, and m6A RNA binding proteins of HMEC cells cultured in Normoxic or Hypoxic conditions for 24 hours and normalized to 28 S rRNA. (N of 3).

A

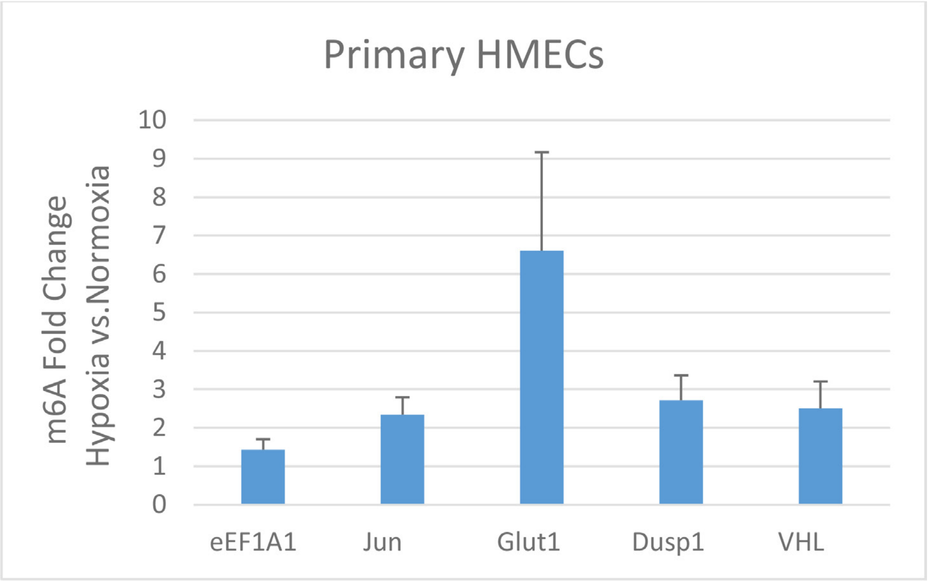

B

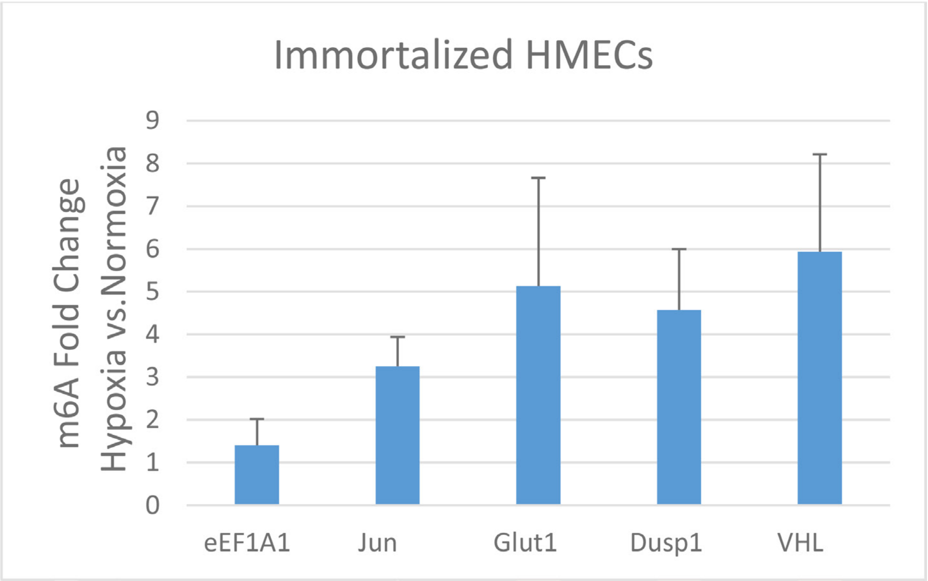

Supplementary Figure 4: m6A IP in primary and immortalized HMECs. MeRIP of 100 ng of mRNA from HMEC primary (**A**) or immortalized (**B**) cells grown in normoxic or hypoxic conditions for 24 hours, and quantified by qRT-PCR. Fold enrichments calculated from immunoprecipitated mRNA levels normalized to input mRNA and expressed as a ratio of hypoxia/normoxia. $^{*}p \leq 0.05$ by paired Student's *t*-test. Error bars represent SEM of 3–4 experiments.

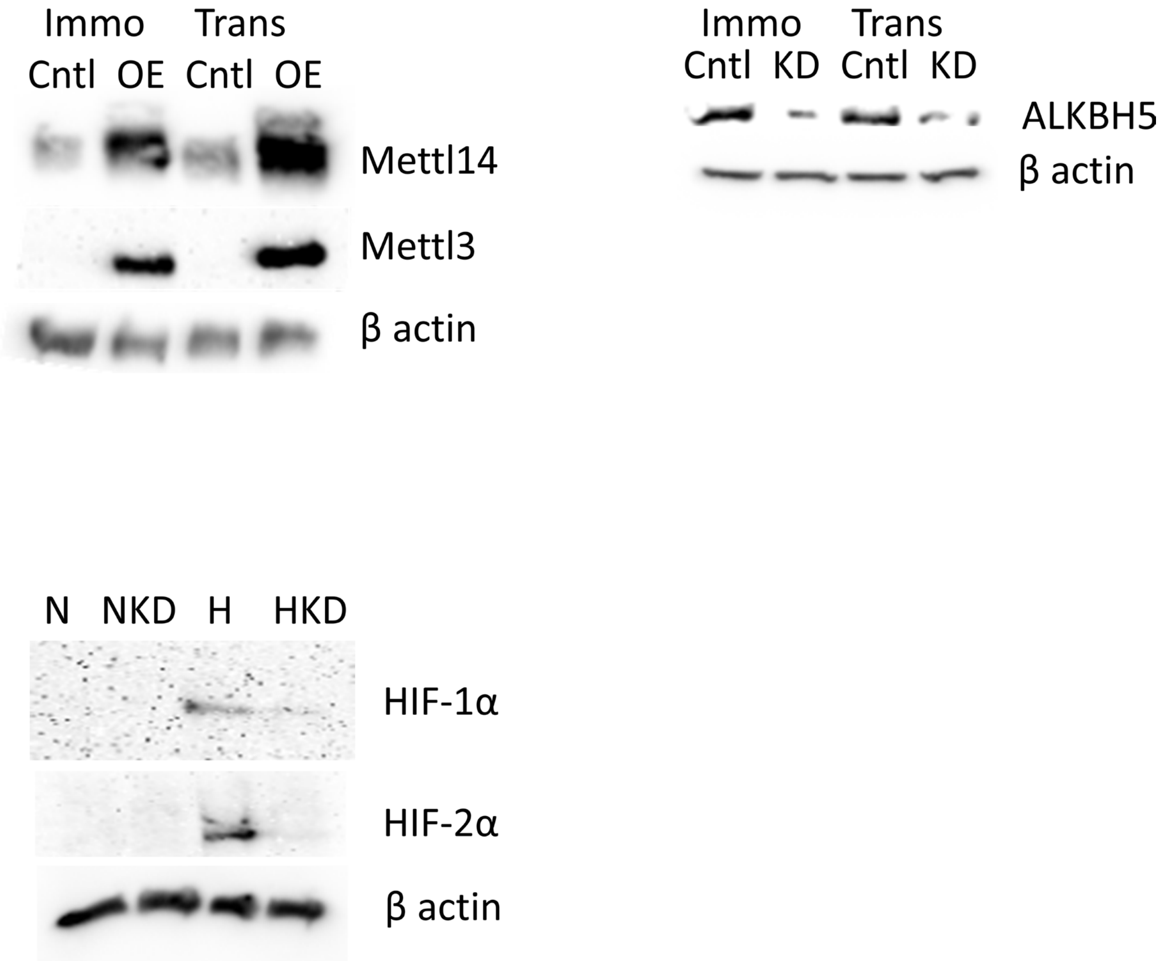

Supplementary Figure 5: Protein levels of Mettl3/14 after overexpression, Alkbh5 after knockdown, and HIF-1 and 2α after knockdown. Western blots of 50 µg of protein lysates of control (Cntl), Overexpression (OE) or Knockdown (KD) of immortalized (immo), and oncogenically transformed (trans) cells. HIF KD blots compare normoxic (N) with or without HIF knockdown (NKD and hypoxic (H) with or without HIF knockdown (HKD).

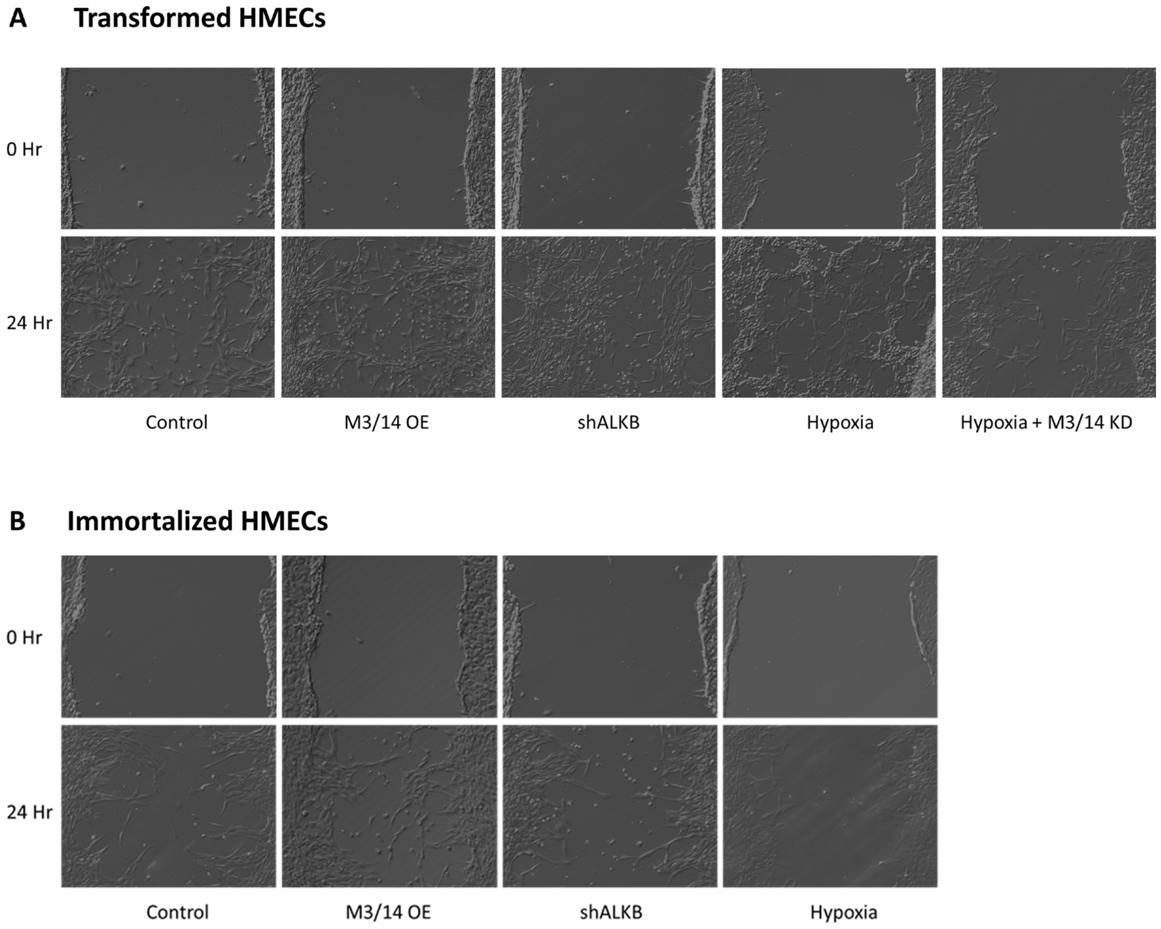

Supplementary Figure 6: Representative scratch assays. Transformed (**A**) and Immortalized (**B**) HMEC scratch assays of negative plasmid transfection (Control), Mettl3/14 Overexpression (OE), ALKBH5 shRNA expression (shALKB), Hypoxia, or Hypoxia plus Mettl3/14 siRNA treatment (M3/14 KD) at 0 and 24 hour time points. (Images representative of 3 experiments).

Supplementary Table 1: Primer list

Gene	Forward (5′-3′)	Reverse (5′-3′)
ALKBH5	GCCTATTCGGGTGTCGGAAC	CTGAGGCCGTATGCAGTGAG
Dusp1	CAACCACAAGGCAGACATCA	CAGTGGACAAACACCCTTCC
eEF1A1	CGGTCTCAGAACTGTTTGTTTC	AAACCAAAGTGGTCCACAAA
FTO	CTGGTTTGGCGATACCCCTT	TTGTGAACCGGCCAAAACAC
Glut1	TATCGTCAACACGGCCTTCACTGT	CACAAAGCCAAAGATGGCCACGAT
Jun	TTCTATGACGATGCCCTCAAC	TCAGGGTCATGCTCTGTTTC
Mettl3	AGCCTTCTGAACCAACAGTCC	CCGACCTCGAGAGCGAAAT
Mettl14	GATAGCCGCTTGCAGGAGAT	CCAATGCTGTCGGCACTTTC
YTHDF1	GCACACAACCTCCATCTTCG	AACTGGTTCGCCCTCATTGT
YTHDF2	AAAGGTCAAGGAAACAAAGTACAAA	TGGGTAAGTAGGAATCTGACATGG
YTHDC1	TCACGGAGGATCTCCTATACAC	GGTAATTCACGCCTGCTGCAAATC
VHL	TCTCTCAATGTTGACGGACAGCCT	GGTCTTTCTGCACATTTGGGTGGT
ZNF217	CCGAAGGAATCCATCTGGATAAA	CCTGTATGCGTTCTGAGATGAA

Supplementary Table 2: siRNA's

siRNA	Product/Sequence
siHIF-1α	Hs_HIF1A_5 S102664053, Qiagen FlexiTube
siHIF-2α	Hs_EPAS1_5 S102663038, Qiagen FlexiTube
siMETTL3	CTGCAAGTATGTTCACTATGA, Qiagen
siMETTL14	AAGGATGAGTTAATAGCTAAA, Qiagen
Negative Control #1 siRNA	Ambion cat. # AM4635

Supplementary Table 3: Antibodies

Antibody	Catalogue #	Vendor	WB Dilution	IP Amount
ALKBH5	HPA007196	Sigma-Aldrich	1/1000	
β Actin	MA5-15739	ThermoFisher	1/5000	
FTO	SAB3500038	Sigma-Aldrich	1/1000	
METTL3/MT-A70	A301-567A	Bethyl Laboratories	1/2000	
METTL14	HPA038002	Sigma-Aldrich	1/1000	
YTHDC1	14392-1-AP	Proteintech	1/200	
YTHDF1	116894	Sigma-Aldrich	1/500	
YTHDF2	Sc-162427	Santa Cruz Biotechnology	1/200	
ZNF217	NBP2-46424	Novus Biologicals	1/1000	
N^6-methyladenosine (m6a)	MABE1006	EMD Millipore		5 µg
